# PKMζ: A Brain Kinase Driving Metabolic Reprogramming and Myofibroblastic Differentiation

**DOI:** 10.1016/j.jcmgh.2024.101437

**Published:** 2025-01-16

**Authors:** Aveline Filliol

**Affiliations:** Department of Cancer Biology and Genetics, Memorial Sloan Kettering Cancer Center, New York, New York

Colorectal cancer (CRC) is a leading cause of cancer-related mortality worldwide, with liver metastases occurring in approximately 25% to 30% of patients, significantly impacting survival outcomes.^1^^,^^2^ Upon colonizing the liver, tumor cells hijack liver-resident cells to create a supportive microenvironment. Among these, cancer-associated fibroblasts (CAFs), primarily derived from hepatic stellate cells (HSCs) in CRC liver metastases, are a major pro-tumoral player.^3^ Upon crosstalk with tumor cells and immune cells, HSCs activate and transdifferentiate into myofibroblastic CAFs, undergoing metabolic reprogramming and producing large amounts of extracellular matrix, cytokines, and growth factors that promote tumor progression and fibrosis.^3^^,^^4^ a process heavily driven by transforming growth factor-β (TGF-β). This transdifferentiation process is metabolically demanding, heavily relying on glycolysis to meet the energy and biosynthetic requirements for cell proliferation and extracellular matrix production. HSC activation is characterized by a metabolic shift, including increased expression of glucose transporters like glucose transporter 1 (GLUT1) and key glycolytic enzymes driving the glycolytic cascade.^5^^,^^6^ Although it is known that CAF is an important tumorigenic driver of CRC metastasis, there is currently no drug that can specifically target them without toxic effect, and targeting HSC metabolism has been suggested as a therapeutic target.^5^

In this issue of *Cellular and Molecular Gastroenterology and Hepatology*, Wang et al^7^ explore how glycolysis drives HSC activation and their transformation into CAF during CRC liver metastasis. They focus on PKMζ, a constitutively active isoform of the atypical Protein Kinase C zeta (PKCζ), previously recognized for its role in the brain but now identified as a key regulator of metabolic reprogramming in HSCs.^8^ The authors show that PKMζ is highly expressed in CAFs within CRC liver metastases and upregulated during HSC activation, both in murine and human primary HSC. Blocking PKMζ, either genetically or using a PKCζ pseudo-substrate peptide, inhibits HSC activation, whereas its overexpression enhances it, highlighting its role in the HSC activation process. Analysis of the HSC transcriptome reveals that PKMζ regulates pathways involved in transcription, metabolism (notably glycolysis), translation, and cell replication, suggesting that PKMζ activity supports the metabolic demands of HSC activation.

Using an array of in vitro and mutational assays, the authors dissect the molecular mechanism by which PKMζ promotes glycolysis. Mechanistically, PKMζ promotes glycolysis by relocating GLUT1, the primary glucose transporter in HSC, to the plasma membrane, leading to increased glucose uptake and ATP production. Interestingly, in a complementary study, the authors demonstrated that specific deletion of GLUT1 in liver fibroblasts using PDGFRβ-CreERT2 mice impaired HSC activation and significantly reduced CRC growth in vivo,^9^ underscoring the importance of GLUT1-mediated glucose metabolism in CAF-mediated tumor progression. Furthermore, the study uncovers how PKMζ forms a complex with vasodilator-stimulated phosphoprotein (VASP) and GLUT1 at the plasma membrane upon TGF-β1 stimulation. PKMζ directly phosphorylates VASP at serine residues S157 and S239, stabilizing GLUT1 at the membrane and amplifying glucose metabolism to meet the energetic demands of HSC activation and CAF function. Finally, the authors used xenografts of human CRC cell lines co-implanted with primary human HSCs, with or without PKMζ knockdown, and observed a partial but significant reduction in tumor growth in nude mice, demonstrating the role of PKMζ in HSC-mediated tumor progression.

The study by Wang et al presents numerous strengths, including the complementary approaches used to dissect the molecular role of PKMζ in the PKMζ/VASP/GLUT1 complex and its regulation of glucose uptake during HSC activation. The extensive use of human material, including primary HSCs and CRC liver metastasis biopsies, enhances the translational relevance of the findings. However, much of the work relies heavily on in vitro experiments, a limitation shared by many studies investigating glucose homeostasis regulation in HSCs.^6^ Cultured HSCs often exhibit enhanced glycolytic activity compared with their in vivo counterparts, potentially overemphasizing molecular dependencies that may not fully translate to physiological conditions.^6^ Future experiments using HSC-specific deletion of PKMζ in vivo would provide critical insights into its functional role under physiological conditions.

Furthermore, an important unresolved question is how PKMζ mediates only a fraction of the TGF-β-regulated transcriptome in HSCs, suggesting that other pathways and regulatory mechanisms may compensate for PKMζ activity. CAFs are a heterogeneous population with distinct spatial distributions and secretomes and are known to exhibit pro- and anti-tumoral functions depending on their context within the tumor microenvironment.^3^^,^^4^ In this context, it is unclear how the glycolytic demands of HSCs, regulated by PKMζ, influence this functional heterogeneity, and understanding the interplay between metabolism and CAF specialization could provide new insights into their dual roles in cancer progression.

In summary, the study by Wang et al identifies PKMζ as a critical regulator of HSC metabolic reprogramming, driving their transformation into CAFs. Future studies should focus on how PKMζ-driven glycolysis shapes CAF heterogeneity and functional duality in cancer progression. Leveraging insights into metabolic reprogramming, it may be possible to design therapies that specifically disrupt tumor-promoting CAF subsets while preserving the homeostatic roles of quiescent HSCs and non-tumorigenic fibroblasts.
